# Natural Deep Eutectic Solvents for PHB Recovery: Mechanistic Insights and Implications for Sustainable Downstream Processing

**DOI:** 10.3390/polym18020169

**Published:** 2026-01-08

**Authors:** Antonio Zuorro, Roberto Lavecchia, Jefferson E. Contreras-Ropero, Janet B. García-Martínez, Andrés F. Barajas-Solano

**Affiliations:** 1 Department of Chemical Engineering, Materials, and Environment, Sapienza University, Via Eudossiana 18, 00184 Rome, Italy; roberto.lavecchia@uniroma1.it; 2 Department of Environmental Sciences, Universidad Francisco de Paula Santander, Av. Gran Colombia No. 12E-96, Cúcuta 540003, Colombia; jeffersoneduardocr@ufps.edu.co (J.E.C.-R.); janetbibianagm@ufps.edu.co (J.B.G.-M.)

**Keywords:** polyhydroxybutyrate, NaDES, extraction, biorefinery, LCA

## Abstract

The growing concern over plastic pollution and the widespread presence of micro- and nanoplastics has renewed interest in polyhydroxybutyrate (PHB) as a biodegradable alternative; however, its industrial deployment remains constrained by costly recovery operations with a high environmental burden. This study examines how PHB biosynthesis and intracellular organization, physicochemical properties, and the characteristics of the producing microorganism influence the performance of conventional recovery routes, including extraction with organic solvents, alkaline/oxidative chemical digestion, and enzymatic–physical schemes coupled with mechanical disruption. Based on this foundation, quantitative data are analyzed for PHB content in bacteria, mixed microbial cultures, cyanobacteria, and microalgae, along with extraction yields, polymer purity, and solvent recyclability in processes employing chlorine-free solvents, green solvents, and hydrophobic natural deep eutectic solvents (NaDESs) formulated with terpenes and organic acids. The analysis integrates mechanistic perspectives on NaDES–cell and NaDES–PHB interactions with solvent design criteria, biorefinery configurations, and preliminary evidence from technoeconomic and life cycle assessments. The findings identify NaDES as an up-and-coming platform capable of reconciling biopolymer quality with the principles of green chemistry while delineating critical gaps in recovery efficiency, viscosity management, solvent recycling, and pilot-scale validation.

## 1. Introduction

The global crisis associated with plastic pollution has emerged as one of the most pressing environmental challenges of the 21st century. The sustained increase in the production of fossil-derived plastics, their low recycling rates, and the persistence of micro- and nanoplastics in aquatic and terrestrial ecosystems have triggered unprecedented regulatory and societal pressures to replace these materials with more sustainable alternatives [1]. Multiple recent analyses agree that improving conventional mechanical or chemical recycling is insufficient on its own and that, in parallel, it is necessary to deploy truly biodegradable bioplastics capable of integrating into biogeochemical cycles without generating long-term cumulative impacts [2].

Within this context, polyhydroxyalkanoates (PHAs) are among the most promising families of biopolymers. PHAs are intracellular polyesters produced by a wide range of microorganisms as a carbon and energy storage mechanism; their biodegradability under environmental conditions and biocompatibility make them particularly attractive for packaging, agricultural, and biomedical applications [3]. Among PHAs, polyhydroxybutyrate (PHB), a homopolymer of 3-hydroxybutyrate, has historically served as a model system. Bacteria such as *Cupriavidus necator* and various *Pseudomonas* species can accumulate PHB at levels of 70–90% of their cellular dry weight under conditions of carbon excess and nutrient limitation, turning these microorganisms into true “bioplastic biofactories” [4]. PHB combines biodegradability, biocompatibility, and mechanical properties comparable to those of certain thermoplastics, such as polypropylene, albeit with higher crystallinity and brittleness, which has motivated both fundamental research and industrial scale-up efforts [5].

However, despite its potential, the commercial deployment of PHAs—and PHB in particular—remains limited. Various technoeconomic assessments indicate that PHA production costs recurrently exceed those of conventional fossil-based plastics, even when inexpensive or residual feedstocks are used [6]. A significant portion of this cost overrun does not originate from fermentation but rather from downstream processing—namely, the recovery, extraction, and purification of the polymer from microbial biomass. Recent reviews addressing the transition of PHAs from the laboratory to the industrial scale emphasize that downstream optimization is decisive for both economic and environmental feasibility, accounting for 30–50% of total production costs, depending on the extraction technology employed [7].

Traditionally, PHB recovery has relied on organic solvent extraction, particularly chlorinated solvents such as chloroform or dichloromethane, which selectively dissolve the polymer from dried biomass and allow subsequent precipitation with an antisolvent. These methods provide high yields and PHB purities near or above 95% and are therefore considered the laboratory-level “gold standard” [8]. However, the extensive use of chlorinated solvents poses severe issues related to toxicity, flammability, volatile organic compound (VOC) emissions, hazardous waste generation, and the need for robust solvent recovery systems. Cost analyses estimate that solvents alone can account for up to ~20% of PHB production costs, even when partial recycling is included [9]. Moreover, the reliance on petrochemical, energy-intensive solvents undermine the environmental claims of bioplastics and complicates regulatory approval in sensitive applications (food, biomedical).

In response, a wide range of alternative extraction strategies have emerged in recent years: alkaline and oxidative treatments, surfactant use, enzymatic digestion of biomass, physical methods (sonication, homogenization, hydrodynamic cavitation, microwaves), and even biological approaches such as insect or microbial consortium digestion [10]. Although some of these methods can reduce the toxicity associated with chlorinated solvents and, in some cases, better preserve the polymer’s molar mass, they often generate saline or chemically complex waste streams, require long residence times, or fail to achieve yields and purities compatible with industrial needs. Consequently, none of these alternatives has succeeded in establishing itself as an integrally superior option to conventional solvent-based routes [11].

Within this framework, deep eutectic solvents (DESs), specifically natural deep eutectic solvents (NaDESs), have attracted increasing attention as “green” extraction media for biopolymers. DESs are mixtures of a hydrogen bond acceptor (HBA) and a hydrogen bond donor (HBD) that exhibit a melting point far lower than that of the pure components, forming a liquid at moderate temperatures owing to an extensive hydrogen-bond network [12]. When these components are metabolites or naturally derived compounds (organic acids, sugars, amino acids, terpenes, choline, etc.), the system is classified as NaDES, characterized by low volatility, reduced toxicity, and potentially high biodegradability, aligning with the principles of green chemistry [13]. Unlike conventional solvents, NaDES can be tailored “on demand,” with polarity, hydrophobicity, and viscosity modulated by selecting HBA/HBD components and their molar ratios. This tunability has enabled the successful extraction of bioactive metabolites, lipids, and aromatic compounds from plant and food matrices [14].

Over the past five years, the use of hydrophobic NaDESs based on terpenes (menthol, thymol, eugenol, etc.) and fatty acids, as well as other natural HBDs, has been systematically explored for the extraction of hydrophobic polymers, including PHAs and PHBV copolymers [15]. Recent studies have shown that certain NaDESs can efficiently solubilize PHB and PHBV at moderate temperatures, achieving recovery yields above 80% and polymer purities in the range of 90–95%, with the additional advantage that NaDES can be recycled over multiple cycles with minimal loss of efficiency in “zero-waste” extraction schemes [16]. These findings suggest that NaDES may represent a realistic alternative to chlorinated solvents, reducing the environmental burden of downstream processing and potentially improving economic feasibility.

Nevertheless, the field remains nascent and fragmented. Although recent reviews address PHA production and recovery, industrial deployment, and related topics [2,17], there is still no synthesis that explicitly focuses on the coupling between PHB and natural deep eutectic solvents (NaDES), their interaction mechanisms, comparative extraction performance relative to conventional and green solvents, and their integration into biorefinery schemes supported by technoeconomic and life cycle assessment frameworks. Importantly, the experimental literature specifically addressing PHB recovery using NaDES remains limited, reflecting the emerging nature of this research area. As a consequence, available studies are currently restricted to a small number of representative hydrophobic NaDES formulations, which nevertheless provide valuable proof-of-concept evidence and mechanistic insight.

Within this context, the present review provides an integrated analysis of PHB’s biological basis, the performance of established recovery routes, and the specific contribution of hydrophobic NaDES to downstream processing. Rather than aiming at an exhaustive compilation of experimental case studies, this work emphasizes the mechanistic foundations, solvent-design principles, and process-level implications that define NaDES as a tunable platform for PHB recovery. Based on this framework, extraction efficiency, polymer quality, solvent recyclability, and integration into biorefinery configurations are critically discussed in comparison with conventional solvent-based and chlorine-free routes, thereby delineating both the current potential and the key limitations that must be addressed for future industrial implementation.

## 2. PHB: Biosynthesis, Properties, and Conventional Recovery Routes

### 2.1. PHB Biosynthesis

From a biochemical perspective, polyhydroxybutyrate (PHB) biosynthesis is organized into a short but highly regulated pathway that links central carbon metabolism to the formation of a storage polymer. The immediate precursor is acetyl-coenzyme A (acetyl-CoA), which is derived from glycolysis, fatty acid catabolism, or anaplerotic routes, depending on the organism and the carbon source used. Two molecules of acetyl-CoA are first condensed by a β-ketothiolase (PhbA) to form acetoacetyl-CoA [18]. This intermediate is then reduced to (R)-3-hydroxybutyryl-CoA by an NAD(P)H-dependent acetoacetyl-CoA reductase (PhbB), and finally, a polyhydroxyalkanoate synthase (PHA synthase, PhbC) catalyzes the polymerization of the monomer, generating PHB chains with molar masses that can exceed 10^5^–10^6^ g·mol^−1^, depending on the strain and cultivation conditions [19].

PHB accumulation is not determined solely by the presence of the enzymatic pathway but also by a deliberate imbalance between the carbon source and limiting nutrients. In many heterotrophic systems, multistage cultivation strategies or fed-batch methods are designed to separate a growth phase (balanced C/N ratio) from an accumulation phase, in which excess carbon is maintained. At the same time, nitrogen, phosphorus, or oxygen are limited [20]. Under these conditions, the cell redirects acetyl-CoA flux from the tricarboxylic acid (TCA) cycle toward the PHB pathway, driven by high availability of reducing power and by specific regulators that coordinate carbon storage. In *C. necator*, an excellent model organism, PHB contents of 80–90% of dry cell weight has been reported in well-optimized fed-batch processes while simultaneously maintaining high cell densities and productivities above 1 g·L^−1^·h^−1^ [21].

In addition to *C. necator*, other Gram-negative bacilli, such as *Paraburkholderia sacchari*, have shown remarkable capacities to accumulate PHB from complex sugar mixtures or streams derived from lignocellulosic biomass. In fed-batch cultures with glucose, xylose, and arabinose mixtures, *P. sacchari* DSM 17165 can reach PHB contents of approximately 70–77% of dry cell weight, with sugar yields of 0.3–0.35 g PHB·g^−1^ [22]. Similarly, various *Bacillus* and *Priestia* spp. (formerly grouped within the genus *Bacillus*) have been evaluated as PHB producers using agro-industrial residues; in some cases, polymer fractions of 40–60% dry cell weight have been reported, making them potentially attractive platforms when the use of residual or low-cost substrates is prioritized [23].

In parallel, mixed microbial cultures (MMCs) selected under “feast–famine” regimes have become established as a relevant platform for producing polyhydroxyalkanoates (PHAs), particularly when the objective is to valorize wastewater streams or complex organic residues. In these configurations, the consortium is enriched with populations capable of storing polymers derived from volatile fatty acids (VFAs) or fermented mixtures, typically achieving PHA contents of 30–70% dry cell weight. It can operate under saline conditions or with variable feed compositions [24,25]. Although the polymer yield per unit biomass is usually lower than that in highly optimized pure cultures, the ability to use waste streams and the associated operational flexibility strengthen their relevance in territorial biorefinery schemes and wastewater treatment.

PHB biosynthesis in photoautotrophic organisms introduces a different scenario in cyanobacteria, such as *Synechocystis* sp. PCC 6803, PHB accumulates under nutrient stress (nitrogen or phosphorus limitation) combined with inorganic carbon excess, which makes the PHB pathway a carbon sink coupled with oxygenic photosynthesis. In wild-type strains, the PHB content rarely exceeds 10–15% dry weight; however, metabolic and regulatory engineering strategies, including the overexpression of PHB genes and regulators such as PirC, have yielded strains with PHB contents of up to ~80% dry cell weight under photoautotrophic and mixotrophic conditions [26]. Complementary screening studies in multiple cyanobacterial strains have shown more modest PHB contents (generally <10% of dry weight), as in *Nostoc muscorum* or *Spirulina (Arthrospira) platensis*, but with the advantage of operating in open or semiclosed systems fed with CO_2_ [27]

In eukaryotic green microalgae, strategies have been developed to introduce the PHB pathway de novo, leveraging its ability to fix CO_2_ and its established use in bioremediation and high-value metabolite production. In *Chlamydomonas reinhardtii*, the insertion of bacterial phbB and phbC genes from *C. necator* enabled the demonstration of intracellular PHB granule formation, initially with low polymer fractions, which later increased through peroxisomal relocalization of the pathway and optimization of acetyl-CoA fluxes [28]. Similarly, appreciable PHB contents have been reported in *Spirulina platensis* and *Hematococcus pluvialis* under stress conditions combined with acetate supplementation, showing that polyester accumulation can be integrated with pathways already exploited for pigment and lipid production [28]. Table 1 summarizes a representative set of these microorganisms and consortia, indicating their metabolism, cultivation conditions, and the maximum reported PHB content.

This diversity of producers (Table 1) translates into highly distinct cellular architectures that directly determine extraction requirements. Gram-negative bacteria such as *C. necator* or *Paraburkholderia* possess an envelope with an outer membrane and a relatively thin peptidoglycan layer, whereas *Bacillus* spp. exhibit thicker, peptidoglycan-rich cell walls, which typically require more aggressive lytic agents. In cyanobacteria and microalgae, PHB granules are located within a more complex organizational context, including thylakoids, vacuoles, and extracellular polysaccharide matrices that act as diffusion barriers; consequently, polymer release requires combinations of osmotic stress, thermal treatments, or physical aids (e.g., ultrasound, homogenization). In mixed cultures, ultimately, the polymer is distributed among multiple cell populations with walls of varying composition and is frequently embedded within flocs rich in extracellular polymeric substances (EPSs), adding a layer of heterogeneity to downstream process design.

### 2.2. Physicochemical Properties Relevant to Extraction

The intrinsic properties of polyhydroxybutyrate (PHB) determine both its behavior during processing and the choice of recovery method. PHB is a highly crystalline polymer (typically 60–80% crystallinity) with a melting temperature of approximately 170–180 °C and a glass transition temperature close to 0 °C [5]. This high crystallinity underpins mechanical properties comparable to those of certain fossil-based thermoplastics but also leads to brittleness and a tendency to crack upon aging (as crystallinity increases over time). From an extraction standpoint, the crystallinity and hydrophobic nature of PHB account for its insolubility in water and most polar solvents, as well as its affinity for organic solvents of intermediate to low polarity, such as halogenated solvents (chloroform, dichloromethane) or certain esters and organic carbonates at elevated temperatures [9].

Another critical aspect is the polymer’s thermal and chemical stability. PHB undergoes random chain scission when exposed for prolonged periods to temperatures near or above its melting point, which results in a reduction in molar mass and, consequently, deterioration of its mechanical properties. Similarly, exposure to oxidants (e.g., hypochlorite) or strongly alkaline media can degrade the polyester chain, even when the polymer does not fully dissolve [10]. Therefore, extraction methods must strike a balance between conditions that are sufficiently intense to release and solubilize PHB yet mild enough to avoid significant degradation.

The biodegradability and biocompatibility of PHB do not directly affect the extraction step, but they strongly influence purity requirements and limit the presence of residual contaminants. In biomedical or food-contact applications, the presence of trace amounts of toxic solvents, residual surfactants, or degradation products may be unacceptable, necessitating more stringent purification processes [5]. In this context, choosing extraction systems that employ low-toxicity solvents (such as natural deep eutectic solvents (NaDESs) formulated from food-grade or pharmaceutical compounds) can facilitate regulatory acceptance and reduce the extent of subsequent purification.

### 2.3. Conventional PHB Recovery Routes

**Figure 1:**
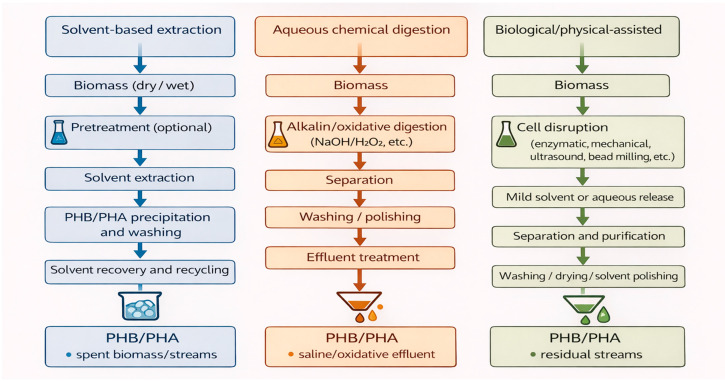
Industrial Downstream Strategies for PHB Purification.

From the perspective of PHB downstream processing, virtually any industrial process converges toward variants of three basic operational families, represented in Figure 1: processes based on extraction with organic solvents, routes involving cell lysis and chemical digestion in aqueous media, and strategies that combine enzymatic methods with physical disruption of the cell followed by separation and washing steps for the polymer. Several reviews dedicated to PHA recovery concur that these three approaches encompass most experimental and pilot-scale developments and that each occupies a specific niche within process design: solvent-based routes stand out for achieving high purity and preserving polymer molar mass [17]. Chemical digestion schemes prioritize operational simplicity and moderate costs, even at the risk of partial PHB degradation [32]. In contrast, enzymatic–physical approaches aim to maximize the environmental compatibility and structural preservation of the polymer but require tighter control of operating conditions and equipment [32].

(a) Extraction with Organic Solvents

Extraction with organic solvents remains the benchmark when the primary objective is to obtain PHB of very high purity with minimal chain degradation. In the scheme depicted in Figure 1, PHB-rich biomass is harvested, conditioned (typically by drying or lyophilization), and brought into contact with a solvent capable of selectively dissolving the polymer (chloroform, dichloromethane, 1,3-dioxolane, ethyl acetate, or dimethyl carbonate, among others). The undissolved solids are then removed by filtration or centrifugation, and PHB is recovered by precipitation with an antisolvent or by controlled solvent evaporation [17].

Classical experiments with *C. necator* revealed that chloroform extraction can recover PHB with purities above 97% and molar masses on the order of 10^6^ g·mol^−1^, which explains its widespread use as the laboratory “gold standard.” A representative example is the work of Fiorese et al., who combined hypochlorite and chloroform to obtain polymers with purities close to 97% and molar masses comparable to those of the original, while minimizing degradation during treatment [8].

On this basis, fewer hazardous solvents have been proposed. The use of 1,3-dioxolane as a “green solvent” enabled the recovery of PHB and PHB-co-HV copolymers with recoveries above 90% and purities close to 99%, while maintaining good processability of the polymer and demonstrating scalability from a few milliliters to liter-scale volumes [33]. Similarly, the use of dimethyl carbonate (DMC) as the primary solvent and ethanol as a polishing agent has yielded extraction yields comparable to or higher than those with chloroform, with clear advantages in terms of toxicity and environmental footprint, as studied by [34]. The life cycle assessment by [35] reported significant reductions in impacts associated with emissions and waste management.

Despite these advances, systematic comparisons among solvent extraction routes show that most processes remain solvent- and energy-intensive, and that solvent recovery and recirculation largely determine economic and environmental feasibility at the industrial scale. A recent comparative review of PHA extraction processes from mixed cultures revealed that the best results in terms of purity and polymer properties are obtained with chloroform, but at the cost of higher operating expenses and environmental risks than those associated with nonhalogenated solvents [17].

(b) Lysis and chemical digestion in aqueous media

The route illustrated in Figure 1 involves resuspending biomass in an aqueous medium and selectively degrading the non-PHA fraction with chemical agents, leaving PHB granules as a concentrated solid phase. The best-known example is digestion with sodium hypochlorite, which oxidizes proteins, nucleic acids, and lipids, enabling the isolation of PHB with high purity, albeit at the cost of substantial degradation of the polyester chain and, therefore, a reduction in molar mass [36]. A previous study [3,32] showed that hypochlorite treatment in the absence of solvent drastically reduces the polymer molar mass, whereas combining hypochlorite with a chloroform phase mitigates this degradation owing to a “shielding” effect of the organic phase around the polymer.

To reduce the use of strongly oxidizing agents, combinations of alkalis (NaOH, KOH) and surfactants have been explored. Gu et al. demonstrated that PHA recovery from mixed cultures improves when hypochlorite is replaced by a sequence of alkaline digestion and washing, particularly when coupled with green solvents such as DMC in subsequent stages [37]. Other studies have evaluated mixed NaClO/NaOH formulations to characterize the trade-off between extraction yield and polymer degradation in both bacteria and microalgae, defining operating windows that maximize recovery with an acceptable loss of molar mass [38].

From a sustainability standpoint, environmental and cost assessments of downstream operations indicate that alkaline and oxidative digestion schemes can result in operating costs per kilogram of polymer lower than those of solvent extraction. Still, they shift the environmental burden to the effluent treatment line owing to the high salinity and oxidant demand of the generated liquid streams [39]. Thus, chemical digestion has emerged as an attractive option for applications where the molar mass is not critical and where infrastructure is available to handle concentrated saline streams. Still, it is less suitable when high-performance biopolymers are required or when stringent discharge regulations apply.

(c) Enzymatic and physical methods

The processes represented in Figure 1 employ lytic enzymes and physical disruption to release PHB granules with minimal chemical damage. In enzymatic methods, lysozymes, proteases, glucosidases, and other hydrolases are used to selectively degrade the cell envelope and the protein–lipid layer surrounding the granules, allowing the polymer to be recovered by centrifugation or filtration, followed by washing steps. A recent review on the effectiveness of PHA recovery methods highlights that these methods can preserve molar mass to a remarkable extent and reduce the need for aggressive reagents. However, they are limited by enzyme costs, treatment duration, and the need for tightly controlled operating conditions [32].

Physical methods are used both as standalone alternatives and, more frequently, as pretreatment modules coupled with chemical digestion or solvent extraction. Ultrasound, high-pressure homogenization, bead milling, microwaves, and hydrodynamic cavitation have been shown to weaken the cell wall and promote the release of PHA granules. Lad et al. reported that hydrodynamic cavitation applied to bacterial biomass improved subsequent PHB extraction, reducing total processing time and achieving efficiencies of approximately 70% under optimized conditions [40]. More recently, Nayir et al. evaluated PHA recovery from mixed cultures via hydrodynamic cavitation and reported yields above 70% with operation times on the order of minutes and purities of approximately 70%, positioning this technology as a promising candidate for intensified downstream processes [41].

Combinations of ultrasonication with alkaline digestion have also been tested. Bhat et al. showed that prior ultrasonic disruption followed by NaOH treatment improves PHA recovery from mixed cultures and allows operation at more moderate alkali concentrations without loss of yield, opening the door to hybrid configurations in which energy and chemical inputs are more efficiently balanced [42]. Similarly, ref. [36] evaluated P(3HB) recovery by high-pressure homogenization directly from wet biomass and demonstrated that high recoveries can be achieved without resorting to organic solvents, provided that the pressure, number of passes, and subsequent separation sequence are optimized.

## 3. PHB/PHA Extraction Routes: Conventional Solvents, Green Solvents, and NaDES

Efficient recovery of polyhydroxyalkanoates (PHA) requires balancing extraction yield, polymer quality, operator safety, and solvent recyclability. Chloroform-based extraction is still often considered the “gold standard” for PHA recovery: it readily dissolves intracellular PHAs and yields exceptionally high purities [39]. In fact, recent reviews note that solvent-based recovery (e.g., chloroform, acetone) routinely achieves >90% purity. However, chloroform use entails serious drawbacks: it must be applied in significant excess and cannot be fully recycled, making the process costly and toxic [43]. For example, ref. [43] reports that although chloroform can recover >90% of PHB with high purity, it is typically used only once (no reuse) and poses a significant health and environmental hazard. These issues have driven the search for safer, greener extraction routes.

One strategy has been to replace halogenated solvents with benign organic solvents. Ethyl esters have shown great promise. Alfano et al. (2021) demonstrated that among several ethyl esters tested on mixed-culture PHBV biomass, ethyl acetate (EA) was superior: it dissolved the copolymer at the lowest temperature and allowed high-purity recovery [44]. In optimized extraction runs, EA extracts PHBV with purities >95% at milder conditions than other esters, indicating that non-toxic esters can match solvent performance. Likewise, ref. [34] found that dimethyl carbonate (DMC) yields PHB extraction efficiencies comparable to or exceeding those of chloroform. In their study on recombinant *E. coli* biomass, PHB yield with DMC was ≥67% (like chloroform), and polymer purity remained high [34]. Significantly, they also performed a life-cycle assessment showing that replacing chloroform/hexane with DMC/ethanol reduced health impacts. These results suggest that esters and carbonates can effectively substitute for chloroform in PHA extraction, though the process must then focus on efficient solvent recovery and spent biomass handling.

A second approach eliminates organic solvents by using aqueous chemical treatments. For instance, ref. [45] developed “chlorine-free” mixed-culture extractions using alkaline and oxidative digestion. In pilot-scale tests on sludge-derived biomass, sequential NaOH and H_2_O_2_ treatments lysed cells and released PHA with recoveries around 70–88% and purities of 92–93% [45]. These chlorine-free methods avoid hazardous solvents but generate highly saline, oxidant-rich effluent streams. In practice, the alkali/oxidative digestion greatly simplifies equipment (no solvent), but shifts complexity to wastewater treatment: the spent liquor requires neutralization and salt removal, especially at scale. Overall, these chemical methods demonstrate that purely aqueous extraction can achieve high purity and good yield, but environmental and disposal issues must be carefully managed.

Beyond solvents and chemicals, researchers have investigated intrinsically “green” organic media. Cyclic acetals and bio-derived ketones are notable examples. The cyclic acetal 1,3-dioxolane, for example, has been used to replace chloroform in PHB recovery. ref. [33] showed that hot 1,3-dioxolane extraction gives >90% PHB recovery with ~97–99% purity. In their study, dried *C. necator* cells yielded 96.6 ± 0.1% PHB (99.1 ± 0.6% purity) in 80 °C extraction, and even wet cells gave ~94–97% yield/purity [33]. Similar results were obtained on scale-up. Thus, 1,3-dioxolane proves almost as effective as chloroform for PHB, but with far lower toxicity and volatility. Other bio-based solvents have also been tested. For low–molecular-weight PHBV, ref. [46] found that 2-methyltetrahydrofuran (2-MTHF) and dihydrolevoglucosenone (Cyrene) both dissolve PHA well: 2-MTHF achieved ~62% yield of PHBV (≥99% pure) and Cyrene ~57% yield (≥99% pure) under optimized conditions [46]. These green solvents (esters, carbonates, acetals, bio-ketones) all combine good PHA solubility with improved safety. For example, Yabueng et al. (2018) reported that using 1,3-dioxolane and water as the antisolvent yielded ~92.7% PHB recovery at 97.9% purity [47]. Similarly, 2-MTHF and Cyrene have negligible vapor pressure and can be recycled. In summary, careful solvent selection (esters, acetals, bio-derived ethers) can preserve extraction efficiency while drastically reducing hazardous emissions [33,46].

The most recent frontier is the use of natural deep eutectic solvents (NaDES) for PHA recovery. NaDES are mixtures of natural compounds (hydrophobic alcohols, acids, sugars, etc.) that form low-melting liquids. By tuning the HBA/HBD components, a NaDES can be designed to dissolve PHAs selectively while remaining relatively benign and recyclable. Hydrophobic NaDES are especially promising for PHB/PHA. For example, ref. [48] synthesized a thymol:vanillin NaDES (8:2 molar ratio) and used it, with sonication and 1-heptanol as an antisolvent, to extract PHBV from mixed biomass [48]. This process recovered about 42% of the polymer, but with outstanding purity (~99%) and without altering the copolymer composition. Notably, ref. [48] demonstrated that both NaDES and heptanol could be recycled. In parallel, ref. [43] applied an L-menthol:acetic-acid NaDES (1:3) to engineered *Pseudomonas putida* biomass. Their optimized extraction (with methanol as the antisolvent) yielded ~66% PHB at ~85% purity [43], comparable to traditional solvents but using a fully biogenic solvent system. In both cases, NaDES solvents were far less toxic than chloroform and can be recovered. These studies confirm that hydrophobic NaDES can selectively dissolve PHAs: they engage polar (acidic HBD) and nonpolar (terpene HBA) interactions with PHB’s functional groups, while excluding most impurities. The trade-off is typically a slightly lower yield than chloroform, but this “yield gap” is offset by the green benefits of NaDES. For example, Mondal et al. noted that thymol:vanillin NaDES achieved near-quantitative purity with half the yield [48]. In practice, NaDES extraction is often combined with cell-disruption (sonication, enzymes, etc.) to maximize release. The key advantage is that NaDES offers a closed-cycle solvent system: once PHB is precipitated, the eutectic mixture can be reconstituted and reused.

From a solvent-design perspective, the two NaDES systems already validated for PHB/PHA extraction can be more appropriately understood when placed within a significantly broader family of hydrophobic NaDES that has been systematically developed in the green-solvent literature over the last decade. In this context, terpene-based eutectic systems play a central role, as they were initially conceived to combine low water miscibility, reduced viscosity, and tunable hydrogen-bonding strength. Consequently, these systems define a physicochemical space that closely matches the requirements for selective PHB solubilization. Within this family, thymol-based NaDES have emerged as particularly versatile, since combinations such as thymol:coumarin (2:1 and 1:1), thymol:menthol (1:1 and 1:2), and menthol:1-tetradecanol (1:2) consistently exhibit stable liquid formation, low polarity, and pronounced hydrophobic character [49]. As a result, these properties favor interactions with the PHB backbone while simultaneously limiting the co-extraction of polar cellular components.

Building on this terpene-based framework, thymol–fatty acid NaDES introduces an additional and complementary degree of tunability by enabling a systematic increase in hydrophobicity through alkyl-chain length variation [50]. Specifically, eutectic mixtures of thymol with octanoic, decanoic, or dodecanoic (lauric) acid have been reported to exhibit low melting points and adjustable viscosity and phase behavior. In the context of PHB extraction, this structural variability becomes particularly relevant because increasing fatty-acid chain length enhances the exclusion of proteins and carbohydrates while modulating the balance between polymer dissolution and controlled precipitation. This effect is especially crucial under antisolvent-mediated recovery schemes, where solvent–polymer interactions must be carefully balanced to ensure efficient precipitation without excessive solvent loss [50,51].

In parallel, menthol-based NaDES represent another structurally coherent branch within the hydrophobic eutectic landscape. Beyond the L-menthol:acetic acid system already discussed, these formulations have been expanded into broader eutectic libraries specifically designed to combine low viscosity with moderate hydrogen-bond donor strength. Although PHB-specific extraction datasets are not yet available for all menthol-derived NaDES, their demonstrated ability to solubilize hydrophobic biopolymers, lipids, and polyester-like substrates indicates clear technical relevance for PHA downstream processing. Moreover, their low vapor pressure and benign toxicological profile further support their suitability for extraction processes operated under mild thermal conditions and reduced volatile organic compound emissions [52].

Further diversification of hydrophobic NaDES has been achieved through alternative terpene-based combinations, pairing thymol with hydrophobic cyclic molecules such as 1,8-cineole or camphor. In these systems, differences in molecular shape and packing give rise to distinct microheterogeneous liquid structures. Consequently, such structural features influence diffusion processes, swelling of disrupted biomass matrices, and the accessibility of intracellular PHB granules following cell disruption. These effects become increasingly relevant when extraction is carried out from wet biomass or mixed microbial cultures, conditions under which solvent nanostructuration and phase stability exert a dominant influence on mass-transfer kinetics [53].

At the level of process integration, exploratory investigations of hydrophobic NaDES further highlight a set of practical constraints that directly inform PHB extraction design. In particular, water uptake during repeated extraction cycles, solvent-property drift induced by dissolved solutes, and partial partitioning of eutectic components into aqueous phases have been identified as critical factors [54]. Taken together, these phenomena determine whether a given NaDES can function as a truly closed-cycle solvent when integrated with cell disruption, washing steps, and antisolvent-induced polymer recovery. Accordingly, NaDES selection for PHB/PHA extraction is increasingly framed as an optimization problem in which hydrophobicity, hydrogen-bond balance, and resistance to water-induced destabilization must be simultaneously addressed [54,55].

Within this framework, the NaDES formulations that appear most immediately transferable to PHB/PHA extraction are those that combine strong hydrophobic character, a tunable hydrogen-bond donor-acceptor balance, and demonstrated liquid stability under process-relevant conditions. Representative systems include thymol:vanillin (8:2), thymol:coumarin (2:1 and 1:1), thymol:menthol (1:1 and 1:2), menthol:1-tetradecanol (1:2), thymol:octanoic acid, thymol:decanoic acid, thymol:dodecanoic acid, and thymol:1,8-cineole or thymol:camphor mixtures. By explicitly situating these chemically distinct eutectic compositions within the NaDES platform concept, the discussion of PHB recovery can move beyond isolated case studies and toward a structured solvent-design space that supports rational downstream-process development [49].

Accordingly, these trade-offs and representative solvent systems, encompassing conventional solvents, green organic media, and NaDES-based routes, are summarized in Table 2.

Each method shows distinct trade-offs: chloroform yields high recovery and purity but poor sustainability, whereas NaDES systems sacrifice some recovery (∼40–70%) to achieve high purity with non-toxic, recyclable solvents [43,48]. Emerging green solvents such as 1,3-dioxolane or DMC combine the best of both worlds, delivering very high purity and good yields [33,34]. The choice of extraction route thus depends on the application priorities (maximum yield, maximum purity, or environmental impact). In all cases, the trend is clear: moving away from halogenated solvents towards biodegradable, recyclable media (esters, carbonates, acetals, or tailored NaDES) can preserve extraction effectiveness while significantly improving safety and sustainability [44,48].

## 4. Mechanistic Perspectives on NaDES–Cell and NaDES–PHB Interactions

Understanding how a natural deep eutectic solvent (NaDES) interacts with microbial cells and with the polymer PHB at the molecular level provides a basis for rationalizing experimental outcomes and guiding process improvements. With respect to NaDES–cell interactions, several studies suggest that certain NaDESs can destabilize cellular structures [57,58]. For example, unlike inert organic solvents, some NaDESs can partially weaken plant and microalgal cell walls, increasing membrane permeability [59]. Although the peptidoglycan wall and membrane structure of bacteria differ from those of plant cells, analogous mechanisms are expected. A key factor is the NaDES composition: if it is acidic or contains strong organic acids, it may lower the local pH and affect wall/membrane components. Indeed, it has been proposed that an excess of protons (H^+^) from acidic NaDES can adsorb onto the cell membrane surface, altering lipid conformation and phase behavior and even compromising membrane integrity [56]. This acidic environment denatures membrane proteins and weakens peptidoglycan bonds, facilitating lysis or at least creating pores that make intracellular components (including PHB granules) more accessible.

In addition, NaDES containing low–molecular–weight hydrophobic components (e.g., menthol, thymol, and other terpenes) can intercalate into the lipid bilayer. Terpenes are known to be membrane disruptors with antimicrobial activity, increasing membrane fluidity and permeability. In a fungicidal context, a menthol–thymol NaDES has been shown to induce reactive oxygen species (ROS), leading to severe cellular damage, including membrane damage [57]. In bacteria, the insertion of such hydrophobic molecules may create localized stress regions in the bilayer, weakening the cell barrier. When combined with ultrasound, which generates microcavitation, these effects can lead to micro- or macroscopic envelope rupture, releasing PHB granules into the medium. Experimentally, enzymatic or physical lysis often complements NaDES extraction; for example, Pinelo et al. used hypotonic buffers with lysozyme to weaken the walls of *Pseudomonas putida* before extraction [43], and Mondal et al. applied intense sonication to break cells in a mixed consortium [48]. These findings confirm that NaDES alone does not always breach bacterial defenses but, when combined with lytic or mechanical agents, plays an effective role in synergistic cell disruption. Mechanistically, NaDES-mediated PHB release arises from chemical stress (acidic denaturation), the insertion of amphipathic molecules into the membrane, and osmotic dehydration, because highly concentrated eutectic mixtures can extract water from the cell. Although not directly reported, this third effect is plausible, as NaDES strongly sequesters water, potentially causing plasmolysis and facilitating PHB release.

Concerning NaDES–PHB interactions, the focus is on how NaDES dissolves or retains PHBr. PHB is a polyester with carbonyl and methyl groups in its repetitive unit ([-O-CH(CH_3_)-CH_2_-CO-]_n_). Effective dissolution requires interactions with both the polar carbonyl group and the hydrophobic backbone. The hydrophobic NaDES used for PHB extraction (e.g., L-menthol:acetic acid, thymol:vanillin) has dual interactions: the polar hydrogen bond donor (HBD) component (acetic acid, vanillin, etc.) can form hydrogen bonds with PHB carbonyl groups, whereas the apolar organic component (menthol, thymol) establishes van der Waals interactions with hydrophobic regions. This combined polar–apolar solvation capability likely underlies PHB dissolution in NaDES, a property not equally provided by single-component solvents. From a thermodynamic standpoint, the eutectic phenomenon involves charge delocalization and a decrease in entropy of mixing [60], which may create a favorable environment for accommodating macromolecules such as PHAs. Solubility studies have shown that under moderate heating, NaDES can dissolve appreciable amounts of PHB; for example, ~20% (*w*/*w*) PHB was dissolved in a natural solvent (carvacrol, chemically similar to a monocomponent NaDES) within 30 min at 100 °C [60]. With true NaDES, a solubility of up to ~35% (*w*/*w*) has been reported at ~75 °C [48]. Temperature evidently facilitates the disruption of PHB crystallites and enhances polymer diffusion in NaDES, although solubility saturation imposes a limit. Intracellular PHB exists as semicrystalline granules; a NaDES must penetrate and disrupt crystalline regions to extract it. A suitably chosen NaDES, combined with heat or ultrasound, can achieve partial or complete dissolution. However, once saturation is reached, further extraction stops, explaining why some studies cap recovery at 40–60%, likely owing to solubility limits or PHB trapped in unpermeabilized cell fragments.

Another mechanistic aspect is the selectivity of NaDES for PHB over other cellular components. Ideally, an efficient NaDES dissolves PHB without extracting large amounts of proteins, nucleic acids, or other biopolymers, thereby simplifying purification. The reported purities of ~85–99% indicate that hydrophobic NaDES achieve such selectivity [43], essentially dissolving hydrophobic PHB while excluding most polar biomolecules. Even membrane lipids (also hydrophobic) often remain insoluble or segregate into a different phase, as observed when heptanol was used by Mondal et al. for selective separation [48]. This differential affinity arises because eutectic networks are not universally compatible with all solutes, and many large biomolecules cannot be incorporated into the hydrogen-bond network of the NaDES. Moreover, NaDES generally operates at moderate temperatures (30–60 °C), which are insufficient to degrade macromolecules to the same extent as alkaline or hypochlorite treatments, which hydrolyze proteins and release impurities. Consequently, NaDES extraction acts more “benignly,” selectively extracting PHB owing to its polarity compatibility.

**Figure 2:**
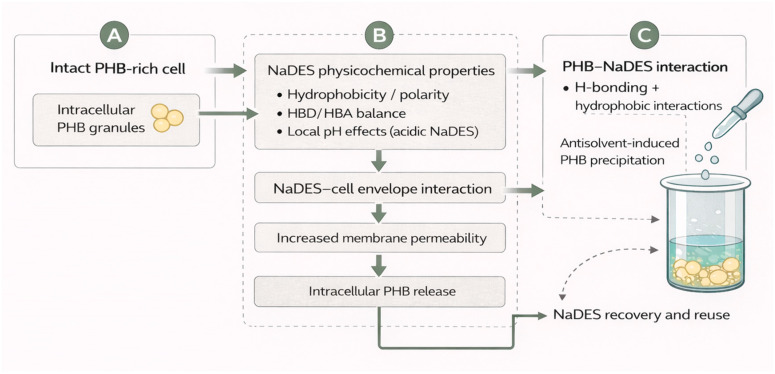
(**A**–**C**) Mechanistic interactions of NaDES with bacterial cells and intracellular PHB.

Finally, the NaDES–PHB–antisolvent interaction governs polymer recovery. When a polar antisolvent (e.g., water or methanol) is added to the PHB-loaded NaDES, the eutectic structure is disrupted, leading to phase separation or an increase in effective polarity, drastically reducing PHB solubility and inducing precipitation. Mechanistically, this step involves competition for hydrogen-bonding sites. Methanol, for example, competes with acetic acid and vanillin for hydrogen bonding, disrupting NaDES cohesion and precipitating PHB due to its poor solubility in the new medium [43]. In contrast, Mondal et al. used an immiscible secondary solvent (heptanol), into which impurities preferentially migrated, leaving PHB in the NaDES phase from which it crystallized upon cooling [48]. Regardless of the method used, understanding these final interactions is key to maximizing recovery: ideally, NaDES selection must consider not only extraction capacity but also the ease of subsequent PHB separation (via condition switching, phase addition, chemical displacement, etc.) (Figure 2).

## 5. Integration into Biorefinery Systems

The integration of natural deep eutectic solvents (NaDESs) into biorefinery schemes is supported by increasing evidence that these solvents enable selective biomass fractionation while facilitating the sequential generation of multiple value-added products within a single process stream. Rather than acting as an isolated extraction medium, NaDES has demonstrated the ability to support configurations in which an initial high-value product is recovered and the resulting solid or liquid residue is subsequently transformed into feedstock for additional biotechnological processes, advancing the system toward comprehensive utilization and minimal waste generation [61] presented a clear illustration of this approach using kinnow orange peel: a tailored NaDES was used to extract high-quality pectin, and the remaining biomass was then fermented with *Lactobacillus amylophilus*, achieving L-lactic acid titers above 55 g L^−1^, thereby establishing a NaDES-assisted citrus biorefinery (ScienceDirect).

Comparable outcomes have been reported in lignocellulosic matrices. Mondal et al. evaluated the disruption of oilcane bagasse via various NaDESs composed of choline chloride and organic acids, achieving the selective solubilization of lignin and hemicellulose. This selective fractionation enabled glucose yields above 80% after enzymatic hydrolysis while significantly increasing the recoverable lipid content [62]. In this case, NaDES served as a biomass fractionation agent, facilitating the recovery of fermentable sugars, lipids, and lignin in separate process streams while remaining recyclable across multiple cycles with minimal solvent loss. These findings reinforce the role of NaDES as a structural, reusable component rather than a disposable input. Together, these examples—in citrus waste and oilcane bagasse—demonstrate that NaDES use is empirically associated with configurations in which biomass is treated as a multiproduct source, and the solvent circulates internally in a reuse cycle.

Based on these findings, current data support the development of a PHB-specific biorefinery in which NaDES plays a role analogous to its own. In a representative scenario, an organic residue (e.g., agroindustrial effluents, low-value sugar streams, or pretreated leachates) is used as a feedstock for a PHB-accumulating bacterial culture optimized to achieve high intracellular polymer content. After fermentation, the biomass is harvested and concentrated via centrifugation or filtration. A hydrophobic NaDES designed to exhibit strong affinity for PHB or short-chain-length PHAs was then applied. Recent literature provides such examples: Mondal et al. employed thymol:vanillin NaDES (8:2) in combination with 1-heptanol, achieving PHA purities approaching 99% despite overall recoveries of only ~42% [62]. Similarly, Didion and Pinelo reported a hydrophobic L-menthol:acetic acid NaDES (1:3) applied to *Pseudomonas putida* biomass, achieving PHB recoveries and purities comparable to those obtained with chloroform but with a markedly more favorable safety and sustainability profile [43].

Within a biorefinery framework, the PHB-loaded NaDES phase is subsequently subjected to a controlled shift (e.g., the addition of a miscible antisolvent such as methanol or ethanol, pH adjustment, or the use of a switchable NaDES), which triggers a loss of polymer solubility and the precipitation of a purified PHB fraction. This mechanism, previously demonstrated for PHA extraction using hydrophobic NaDES, enables the recovery of high-purity biopolymers while retaining the NaDES as a recyclable liquid phase that requires only minimal reconditioning before reuse [62]. The residual solid fraction—composed of cell walls, proteins, polysaccharides, and unextracted material—still has substantial value. Prior experience in citrus matrices and oilcane bagasse suggests that this residue can be directed toward anaerobic digestion for biogas production, additional fermentation steps targeting organic acids or bioproducts, or secondary extraction processes using alternative NaDES formulations to recover pigments, phenolics, or protein fractions [61].

Furthermore, the literature demonstrates that appropriate NaDES formulations can be diluted with controlled amounts of water while maintaining extractive capacity, improving scalability, and enabling solvent recycling rates above 90–95% in pretreatment and extraction processes when paired with simple separation operations (filtration, decantation, partial evaporation) [62]. These findings support the feasibility of incorporating PHB-extracted NaDES into an internal cycle within biorefineries, where the solvent behaves as a circulating resource rather than a single-use consumable. Collectively, the studies by Santra, Raj, Mondal, and Didion experimentally justify an integrated scheme in which PHB production, NaDES-based recovery, and valorization of residual fractions are combined within a unified biorefinery platform, offering clear potential to reduce reliance on halogenated solvents, decrease hazardous waste generation, and increase the value obtained per ton of processed biomass.

## 6. Current Limitations and Research Gaps

Despite their promise, the application of NaDES in PHB extraction presents several technical and scientific limitations that recent studies have begun to document systematically.

First, the extraction efficiency is typically lower than that of conventional solvents. Mondal et al. developed a “sustainable” downstream approach using a hydrophobic thymol:vanillin NaDES (8:2) coupled with 1-heptanol for the purification of biomass containing ~49 wt% PHA. Although the polymer purities approached 99%, the actual recoveries were only ~42%, clearly lower than those achieved with chloroform or other conventional solvents [62]. This “yield gap” is also observed in studies by Didion and Pinelo, in which green solvents or NaDES rarely achieve quantitative recoveries compared with chloroform [43].

Second, the high viscosity of many NaDESs restricts mass transfer. In the work of Mondal et al., the choice of thymol:vanillin was explicitly justified by its relatively low viscosity, which reduces the energy demand for agitation and pumping [63]. However, large portions of the formulation space (particularly a NaDES based on sugars or polyphenols) still exhibit high viscosity, which limits biomass penetration and extraction kinetics.

Third, the long-term recyclability and stability of NaDES remain insufficiently explored. Although [62] demonstrated multicycle reuse with minimal losses, these studies primarily focus on biomass pretreatment rather than biopolymer recovery. A recent meta-analysis by Suthar on NaDES in separation science emphasized that limited recyclability, high viscosity, and instability in the presence of water, temperature fluctuations, or impurities are key barriers to industrial adoption, underscoring the need for low-viscosity NaDES and more robust recycling strategies [64].

Fourth, data on the toxicity, biodegradability, and environmental footprint of specific NaDES formulations are still scarce. Although many NaDESs composed of natural components are assumed to be biodegradable and low-toxicity, the life-cycle of DES/NaDES processes applied to citrus extracts and agro-food residues has revealed that freshwater ecotoxicity can become the dominant impact category if solvent recovery and effluent treatment strategies are not sufficiently optimized [63].

Finally, the lack of predictive design rules and pilot-scale demonstrations creates a gap between laboratory results and industrial implementation. NaDES selection remains empirical, mainly because all PHB extraction studies using NaDES have been conducted at a small-batch scale. This hinders the assessment of hydrodynamic behavior, material compatibility, equipment fouling, and other scale-dependent parameters.

## 7. Future Perspectives and Industrial Potential

Despite current limitations, the medium-term outlook for natural deep eutectic solvents (NaDES) in polyhydroxybutyrate (PHB) extraction is decisively favorable, particularly within the broader context of green chemistry and bioeconomic strategies aimed at decarbonization, phasing out chlorinated solvents, and reducing volatile organic compound (VOC) emissions [65]. A critical avenue for progress involves the rational molecular design of NaDES specifically formulated to maximize PHB solubilization while enabling controlled precipitation. Integrating predictive tools such as Hansen solubility parameters and COSMO-RS, together with molecular simulations and experimental screening, offers a path toward systems that achieve an optimal balance among polarity, hydrophobicity, and viscosity. In this context, “switchable” NaDESs that alter polarity or miscibility upon exposure to stimuli such as CO_2_, pH, or temperature are especially promising, as they can extract PHB under hydrophobic conditions and subsequently trigger polymer precipitation without the need for large antisolvent volumes [64]. Parallel advances may emerge from process intensification strategies. Opportunities include the implementation of countercurrent extraction columns, membrane contactors, or rotating contactors, which could improve solvent economy and mass transfer, and the use of ultrasound or microwave irradiation, which has already been validated in NaDES-assisted extraction of pectin, phenolics, and essential oils, to reduce residence times and enhance yields in PHB recovery [66].

Another strategic perspective concerns aligning NaDES extraction with high-value PHB applications. The ability of systems such as thymol:vanillin NaDES, reported by Mondal et al., to preserve the PHA composition and molecular structure while achieving high polymer purity, suggests that NaDES-extracted PHB could satisfy the strict purity and performance requirements associated with biomedical, pharmaceutical, and advanced material markets, where the polymer value offsets higher extraction costs [48]. Finally, the demonstrated capacity of NaDES to fractionate lignocellulosic materials—such as citrus residues, oilcane bagasse, or flax byproducts—suggests that future biorefineries in which the same NaDES, or compatible families of NaDES, operate sequentially across biomass pretreatment, metabolite extraction, and PHB recovery. This potential convergence indicates that NaDES may evolve from specialized solvents into multifunctional process media that unify several operations within integrated biorefinery platforms (ScienceDirect).

## 8. Environmental and Economic Assessment

The environmental and economic assessment of NaDES-based PHB extraction remains limited, yet it reveals a consistent set of emerging trends. Environmentally, NaDES offers clear advantages over volatile organic solvents, including low vapor pressure, potential biodegradability, and reduced occupational hazards [65]. Nonetheless, life-cycle assessment (LCA) studies of related NaDES/DES processes—particularly those applied to pectin and essential oil extraction from citrus or flax matrices—indicate that the dominant environmental burden does not necessarily correspond to climate change but rather to freshwater ecotoxicity linked to aqueous effluents containing residual solvents and organic solutes [67]. These results emphasize that NaDES systems will only fulfill their “green” promise if processes are intentionally designed to include closed solvent loops, effluent treatment stages capable of removing organic residues, and strategies to minimize water consumption.

Economically, although widely available NaDES components such as choline chloride, glycerol, and common organic acids are relatively inexpensive, other formulations based on phenolic compounds, including thymol or vanillin, are costlier and may pose barriers to large-scale adoption [63]. The extraction yield directly influences economic viability: processes that recover only ~40–60% of the total PHB [48]. Inevitably incur higher polymer costs per kilogram than the solvent routes approach quantitative recovery. Conversely, solvent recyclability in NaDES-assisted biomass pretreatment and pectin extraction reduces the demand for fresh solvent and improves the cost distribution over multiple cycles [62]. Integration of LCA with technoeconomic analysis (TEA) will ultimately define the threshold conditions—minimum extraction yield, number of reuse cycles, and acceptable solvent component cost—at which NaDES-based PHB extraction becomes competitive with conventional solvent systems, representing a likely transition point toward industrial implementation.

## 9. Conclusions

This review shows that the core bottleneck for the industrial deployment of PHB is no longer fermentation but rather downstream processing. Across a wide range of production systems, the costs, energy requirements, and environmental burdens of extraction and purification consistently dominate the overall process, particularly when chlorinated solvents are used. Conventional solvent routes deliver high purities and preserve molar mass. Still, they are structurally incompatible with current regulatory, toxicological, and sustainability requirements, as well as with alternative aqueous, alkaline, or oxidative strategies. However, they compromise polymer quality or shift impacts to highly saline, chemically complex effluents.

Within this context, natural deep eutectic solvents have emerged not as simple replacements for traditional solvents but as a new design space for PHB downstream processing. Hydrophobic NaDES can be tailored in composition, polarity, and viscosity to solubilize PHB and PHBV at moderate temperatures, achieve purities comparable to those of chlorinated systems, and operate in closed or semiclosed solvent loops while being compatible with biorefinery logics in which biomass is fractionated into multiple product streams. Moreover, the current evidence clearly indicates that NaDES-based extraction is not yet a drop-in solution: typical recovery yields remain significantly lower than those of optimized solvent processes, viscosity and mass-transfer limitations increase operational complexity, and long-term solvent stability, recyclability, and effluent management still require rigorous validation, including integrated environmental and economic assessment.

Consequently, the transition from promise to practice for NaDES-assisted PHB recovery will depend on three convergent advances: rational solvent design explicitly targeted to PHB–NaDES interactions and processability; demonstration of scalable, continuous, and highly recyclable extraction schemes embedded in biorefinery configurations; and robust techno-economic and life-cycle analyses that verify real gains over state-of-the-art nonhalogenated solvent and aqueous routes. If these conditions are met, NaDES-based downstream processing has the potential to eliminate a significant barrier to PHB deployment, align bioplastic production with circular-economy objectives, and reposition PHB from a technically mature but economically constrained material to a viable component of sustainable plastic value chains.

## Figures and Tables

**Table 1:** PHB-producing microbial systems.

Microorganism/Consortium	Type of Metabolism/ Main Carbon Source	Cultivation Configuration/Technology	Maximum PHB Content (% Dry Cell Weight)	References
*Cupriavidus necator* H16	Heterotrophic. sugars and C1–C3 organic acids; also, residual streams	Fed-batch in a stirred bioreactor; two-stage strategies (growth/accumulation)	≈80–90%	[20]
*Paraburkholderia sacchari* DSM 17165	Heterotrophic. mixture of glucose, xylose, and arabinose (lignocellulosic origin)	Fed-batch with ternary sugar mixtures. processes targeted to corn or bagasse-derived streams	≈70–77%	[22]
*Bacillus megaterium*/*Priestia megaterium*	Heterotrophic; glucose, glycerol, and lignocellulosic residues	Batch and fed-batch cultures in mineral medium. use of agricultural residues and industrial effluents	≈40–60%	[23]
*Pseudomonas* sp. phDV1 (genetically modified)	Heterotrophic. Winery industry residues	Aerobic bioreactor cultures using grape byproducts as substrate	≈50–70%	[28]
Mixed microbial culture (MMC) in “feast–famine” SBR	Consortium enriched on volatile fatty acids (acetate, propionate, etc.)	Sequencing batch reactor (SBR) with excess/absence of carbon cycles	≈40–70%	[25]
MMC for PHBV from agri-food residues	Mixed consortium. VFAs generated by fermentation of agri-food residues	Pilot-scale reactors with selection strategies and the subsequent accumulation stage	≈50–65%	[29]
*Synechocystis* sp. PCC 6803 (wild-type and engineered strains)	Photoautotrophic. CO_2_ as a carbon source, with possible acetate addition	Photoautotrophic batch and fed-batch cultures. engineering of regulators and PHB genes	≈10–15% (wild-type) up to ≈80% (engineered strains)	[19,26]
*Arthrospira platensis* and *Nostoc muscorum*	Photoautotrophic. CO_2_, nitrogen limitation, sometimes acetate supplementation	Photobioreactors and raceways under nutrient stress (N, P) and/or acetate addition	≈0.5–6%	[27,30]
*Chlamydomonas reinhardtii* (transgenic strains)	Photoautotrophic/mixotrophic. CO_2_ and acetate. expression of bacterial PHBgenes	Photobioreactor cultures. peroxisomal relocalization of the PHB pathway and optimization of acetyl-CoA fluxes	≈1–10% (Depending on construct and conditions)	[28,31]

**Table 2:** PHB Extraction Methods and Performance Metrics.

Extraction Method and Solvents Used	Typical Conditions (Temp/Time/Biomass Type)	PHB/PHA		Solvent Recyclability	Reference
		**Recovery (%)**	**Purity (%)**		
Conventional chloroform extraction	60–80 °C, 1–3 h, dry biomass	80–99	95–99	Not recyclable	[17]
Extraction with ethyl acetate	50–70 °C, 1–2 h, dry biomass	~90–95	~95–98	Partly recyclable	[43]
Extraction with NaDES L-menthol:acetic acid (1:3) + methanol as antisolvent	30–40 °C, ~6 h, pretreated biomass (enzymatic/mechanical lysis)	~60–70	~80–85	Recyclable after precipitation	[43]
Chemical digestion Aqueous NaOH + H_2_O_2_ (“chlorine-free”)	60–90 °C, mixed or pure cultures	~70–88	~90–93	Not recyclable	[45]
1,3-dioxolane	60–90 °C, 0.5–2 h, dry or wet biomass	~90–97	~95–99	Potentially recyclable	[33]
Nonhalogenated ethyl esters (e.g., ethyl acetate, butyl acetate)	80–120 °C, 0.5–2 h, dry or wet biomass	~50–90	≥90	Recyclable in closed systems	[44]
Dimethyl carbonate (DMC)	60–90 °C, 1–2 h, dry biomass	≥60–70	~85–90	Recyclable	[34]
Multistage processes with combinations of green solvents (cyclohexanone, DMC, alcohols, etc.)	90–130 °C, 3–4 h, dry or wet biomass	~50–98	≥90	Recyclable	[56]
Extraction with hydrophobic NaDES (Thymol:vanillin NaDES (8:2) + 1-heptanol/antisolvent)	40–60 °C, wet biomass	~40–50	Up to ~99	Recyclable after precipitation	[48]

## Data Availability

The original contributions presented in this study are included in the article. Further inquiries can be directed to the corresponding authors.
